# Precautionary Principle and Post-Mortem Brain Resuscitation

**DOI:** 10.1007/s12152-025-09605-5

**Published:** 2025-06-20

**Authors:** Brandon Long, David Resnik

**Affiliations:** Department of Philosophy, University at Buffalo, Buffalo, NY, USA; National Institute of Environmental Health Science, Durham, NC, USA

**Keywords:** Moral philosophy, Philosophy of mind, Bioethics, Neuroscience

## Abstract

Recent advancements in brain research have drastically increased the need for serious ethical consideration. *Postmortem* brain research has taken a significant step in the development of BrainEx. The technology can metabolically resuscitate pig brains from pigs that were “clinically dead” for hours. Ethical discourse around organoids ranges from being overly cautious and sensational to highly permissive and skeptical of even minimal consciousness emerging in such a model. Some of these criticisms are allayed in postmortem brains. As such, postmortem brain research presents philosophers and policymakers with a higher risk model of being conscious. The outcome of researchers unknowingly subjecting postmortem brains to negative mental states through research will be termed brain-in-a-vat-world. We will provide some motivation to believe this is a reasonable outcome of proceeding with postmortem brain research. As such, we propose applying the PP to all postmortem brain research. These precautions go beyond previous precautions of brain activity monitoring and the willingness to administer anesthetics to the brains should they appear awake. These precautions aim to avoid potential negative mental states. Such precautions reasonably mitigate the risk of postmortem brain research causing suffering. We recommend several practical precautions. First, anesthetics may prevent any conscious experience at all, cingulotomies prevent painful experiences, and the administration of opioids may prevent boredom or isolation. IRBs should work to determine how these precautions interact with experimental outcomes and how to balance the risk of side effects in light of the experimentation period. The upshot of the article is such PP applications avoid overapplication of the PP, which has occurred in the brain organoid literature. Further, the PP, we argue, provides better guidance to precautionary policy than decision theory, given the difficulties with applying decision theory in medical ethics.

## Introduction

This paper will apply the precautionary principle (PP) to the present and future applications of recent developments in brain research [1]. The target article shows that contrary to the common assumption, brains can be metabolically resuscitated hours post-mortem with fluid perfusion. The study flushed the vasculature of butchered pigs, removed their brains, placed them in an apparatus termed “BrainEx,” and perfused the brain’s vasculature with the BrainEx perfusate. The BrainEx perfusate contains, among other ingredients, the requirements of the metabolic functioning of neurons. The pig brain vasculature remained patent and serviceable for metabolic activity hours after death and for several hours until the termination of the experiment. The brains were monitored for various metrics, and the changes in the perfusate from the afferent contents to the efferent metabolites were used to analyze metabolic activity. Electrocorticography ensured the brains-in-a-vat (BIVs) never achieved the kind of global neuronal activity correlated with consciousness. Precautions were taken to prevent such global activity, with anesthetics ready-to-hand. However, the neurons were not inert, and neuron samples separated from the brain still possessed neuronal firing capability. This makes the brain “alive,” but we must distinguish this from the brain being “conscious.” It is still unknown if consciousness can be restored in such a model, but given the precautions the researchers took, we believe such a risk is at least a reasonable expectation, despite the fact that electrocorticography correlates with mental states quite well.

This is quite an astonishing research model for various reasons. The most surprising is that brain cells can be restored to some functionality hours after supposed brain death. Such a model will allow researchers to investigate brain functioning with precise control and data collection. We will discuss one potential research aim of such a mode later, but the prospects for drug or disease research are high. However, such a model does not come without risks.

Our main worry is that after much research has been completed, we may be inflicting negative (i.e., highly unpleasant, distressing) mental states on postmortem brains. Mental states are subjective experiences that comprise consciousness and are variegated. For instance, emotions, including fear, disgust, and love; cognitions like retrieving a memory, planning the future, and concentrating on a task; and perceptions such as facial, color, and sound perception [2].

In such a case of uncertainty, the PP seems justified. After Putnam [3], we will coin the undesirable world where research brains are subjected to negative mental states as the brain-in-a-vat world (BIVW). In BIVW, brains-in-vats (BIVs) are subjected to negative mental states unbeknownst to us.^1^ While Putnam popularized the thought experiment to examine skeptical arguments relating to our own experience, we will be using BIVW to raise skepticism about the mental states of the BIVs. Specifically, BIVW invokes skepticism about whether such BIVs are conscious without our knowing it. BIVW raises skepticism about our theoretical knowledge of consciousness and our best experimental data.

We believe the practical precautions outlined in section Conclusion fall out of applying the PP to manage the risk involved in postmortem BIV research. These precautions are anesthetics to prevent consciousness, cingulotomies to prevent pain, and potentially opioids to prevent mental states such as boredom or the feeling of isolation. Adopting such precautions as a groundwork for BIV research may lead to slower scientific progression. Still, the price may be justified, given the significant risk.

Comparative decision theory approaches such as maximin would not permit such research and thus be overly restrictive.^2^ This is the case because the risk of ending up in BIVW is the worst possible outcome, and yet can not be adequately managed via maximin presently. Maximin advises against such brain research, which is overly restrictive. It is overly restrictive, especially considering the additional precautions we advise. Further, our application of the PP for postmortem brains avoids what some researchers have called the overapplication of precaution concerning brain organoids [4]. More generally, our PP does risk slowing research and increasing research expenditure on precautions, but in this specific application, we believe, properly manages the risks and permits research to move forward. Such minimally costly precautions, which hedge against great risk, do not appear to us to be an overapplication of caution.

First, in section Postmortem BIVs a Significant Leap Forward, we will discuss how relevant BrainEx is. In section Traditional Theories of Consciousness, we will discuss theories of consciousness, which help us understand the reasonable precautions in such research. In section Precautionary Principle, we will cover our interpretation of the PP. Section Why the PP and not Expected Utility Theory or Maximin covers why the PP fares better at handling the risks of BIV research. Lastly, section Conclusion will provide some practical precautions that raise the floor and ceiling of potential harm in BIV research and situate the role of IRBs in postmortem BIV research.

## Postmortem BIVs a Significant Leap Forward

The target article [1] utilized the BrainEx system to restore and maintain microcirculation and cellular viability in postmortem pig brains under ex vivo normothermic^3^ conditions. The surgical procedure involved isolating the common carotid arteries for flushing the pig’s vasculature and a craniectomy to expose the brain for removal. The perfusion system included a centrifugal pump, pulse generator, haemodiafiltration system, and gas-infusion mixers. The BrainEx perfusate was a hemoglobin-based, acellular, echogenic, and non-coagulative solution designed to support the brain’s energy requirements and prevent apoptosis. The experiment was 10 h, with a 4-h period before perfusion and a 6-h duration of perfusion. Temperature control and monitoring of global electrical activity were maintained throughout the experiment. Analysis and measurements included high-resolution micro-CT and MRI scans, histology and immunohistochemistry, electron microscopy, and electrophysiology.

The study demonstrated that restoring and maintaining microcirculation and metabolic cellular functions in pig brains hours after death was experimentally viable. Key findings included preserving brain structure, reducing cell death, restoring vascular responses, synaptic activity (a small number of in vitro neurons), and neuronal metabolism. These results suggest that the brain has a greater capacity for recovery after prolonged anoxia or ischemia than previously thought, providing valuable insights into brain resilience and potential applications for brain injury therapies. However, the present and future prospects of research ethics in such a model raise immediate questions. The first question aimed at the present research is how confident we are that metabolically active but synaptically inactive brains have no experience. The second, aimed at future postmortem brain research models, is what kind of brain activity we can reasonably believe indicates a lack of consciousness. The risk of ending up in BIVW is higher for experiments involving neuronal synaptic activity, not just metabolic activity. Still, the PP recommends further caution in both instances, but with differing degrees. This is because it is more reasonable to believe brains with synaptic activity are more likely to be conscious than brains with only metabolic activity.

Other authors have laid out further research ethics questions to help balance risks and rewards. For instance, one article has proposed two helpful questions [5] on brain organoids and consciousness. The first question is simply whether the model is conscious. The second is to elaborate on the kind. Postmortem BIV research presents a significant leap forward in answering the first question affirmatively compared to brain organoids. What evidence do we have to suggest postmortem brains can have subjective experiences in the right conditions? While no reports of this occurring during experimentation have been reported, some neurons of the pigs separated from the whole brain were able to achieve action potentials [1]. This does *not* mean that global activity could be restored to the brains in the experiment, and the potential of this remains unknown.

Further, since postmortem BIVs were previously conscious, this increases the evidence of their being capable of consciousness. Brain organoids present a de novo problem of consciousness. In other words, the assessment of their consciousness comes with no previous evidence of their having been so [4]. Brain organoids thus face a more difficult first question. Postmortem BIVs, conversely, have been as close to verifiable consciousness as we could expect without answering the problem of other minds. Postmortem BIVs attain all the specific brain morphologies (i.e., connections, sizes, specific regions) that correlate with consciousness. Brain organoids presently do worse on all accounts [4].

A lack of sensory inputs creates doubt that postmortem brains or organoids can become conscious. However, there are two different facets to the claim that sensory input is required for consciousness. The first is that sensory input is necessary for all consciousness. Second, sensory input is needed to develop a brain capable of consciousness.

These two interpretations have fundamentally different implications for postmortem BIVs versus organoids. Postmortem BIVs are incapable of consciousness on the first interpretation of the claim, that ongoing sensory input is necessary for all consciousness. However, this interpretation may be too restrictive, given that”islands of awareness”in neural networks disconnected from typical sensory pathways [6] seem plausible. Such islands suggest that consciousness might persist in neural architectures even without current sensory input, particularly when those architectures were developed through normal sensorimotor integration.

Crucially, postmortem BIVs undergo complete development with rich sensorimotor experience, creating neural networks shaped by years of integrated sensory processing. This developmental history may be more important than current sensory access for consciousness. In contrast, organoids face both developmental and current sensory deficits—they develop in artificial environments without the sensorimotor integration that normally guides neural architecture formation, and they lack ongoing sensory input during any potential conscious states.

Therefore, organoids are incapable of consciousness on both interpretations unless we can replicate not only the sensory input essential for brain development, but also the integrated sensorimotor experiences that shape conscious-capable neural architectures. Postmortem BIVs, having already undergone this developmental process, may retain consciousness potential even without current sensory input, making them more likely candidates for artificial consciousness than organoids that never experienced normal neural development. Sensory inputs, therefore, seem vital in either interpretation for the formation of typical human brain morphology [7, 8].

Organoids may even be only capable of creating non-human brain morphologies. It is unclear if such nonhuman morphologies would be capable of consciousness [4]. However, such morphologies would be less translational than postmortem BIVs. It is possible, in principle, to go from our limited ability to provide organoids with input [9, 10] to giving organoids the required sensory inputs for developing into human morphologies. However, postmortem BIVs avoid this need because they were constructed with typical human sensory inputs. In short, we should not discount the leap postmortem BIVs take over brain organoids. Thus, postmortem BIVs are more likely to be conscious and thus present a larger risk than brain organoids. Perhaps current sensory input is also required for consciousness, but on this, organoids and postmortem brains fall short similarly. Presently, research methods are limited in providing such sensory input.

We should mention a few critical areas where BrainEx may be applied in research for potential scientific breakthroughs. Here, the strengths of BIVs are evident when compared to current organoids. Disorders of consciousness likely involve many brain regions, which organoids can not provide. One very plausible application of BrainEx is for Alzheimer’s research, as blood flow and neuronal activity interest Alzheimer’s researchers [11]. This is due to the tight correlation between cerebral blood flow and neuronal activity. Neuronal cell activity causes shifts in blood flow, partially a response to higher O2 demand. Indeed, the researchers in the target article [1] caused vasodilation with merely a metabolically alive model, which shows vascular cells remain active. However, the decoupling of neuronal activity and blood flow is associated with Alzheimer’s [11].

## Traditional Theories of Consciousness

I will briefly cover traditional theories of consciousness and their implications for ethical postmortem brain research. There are three I will cover, which have many variants, but the three broadly cover much traditional theory.

### Higher-Order Theories

Higher-order theories posit that a mental state becomes conscious when it is the object of a specific type of meta-representation—a representation about another representation [12]. For example, the meta-representation “I see a blue cat” pertains to the individual’s internal meta-representational mental state rather than the external environment. This emphasis on meta-representation underpins the explanatory scope of higher-order theories. Consciousness arises when higher-order brain networks redescribe lower-order representations, effectively creating a “meta-representational” state.

The neural basis of higher-order theories often points to anterior cortical regions, particularly the prefrontal cortex [13, 14]. Rosenthal’s [15] higher-order thought theory specifically implicates the dorsolateral prefrontal cortex in generating the metacognitive representations that render first-order states conscious. Similarly, Carruthers’[16] higher-order representation theory emphasizes the role of working memory systems, primarily housed in prefrontal regions, in maintaining the representational states necessary for consciousness. These areas are associated with complex cognitive functions such as self-awareness, confidence judgments, and error monitoring, capacities that align with the meta-representational requirements of higher-order theories [17].

Empirical support comes from neuroimaging studies showing increased prefrontal activity during conscious perception compared to unconscious processing [18, 19]. However, debates persist regarding which specific regions are required for consciousness. Some researchers point to the role of parietal regions in maintaining the global accessibility of higher-order representations [20]. Critics also question whether prefrontal activity reflects consciousness itself or merely the cognitive processes that accompany conscious experience [21].

### Global Workspace Theories

Global workspace theories conceptualize consciousness as a process where information is “broadcast” across a globally accessible neural workspace [12]. Baars’[22] original theory drew inspiration from”blackboard”architectures in artificial intelligence, where a centralized platform enables specialized processors to share and integrate information. This framework proposes that consciousness emerges when sensory information wins a competition for access to the global workspace, allowing it to be broadcast widely throughout the brain [23], highlighting the importance of long-range communication between cortical regions. This integration spans attention, memory, and decision-making processes, with conscious access occurring through amplification, sustaining, and enhancing neuronal representations.

Dehaene and Changeux’s [20] provided the neurobiological implementation, proposing that consciousness corresponds to the global ignition of a distributed cortical network. Their model emphasizes that sensory information becomes conscious when it crosses a threshold and triggers widespread cortical activation, particularly involving long-range cortico-cortical connections [24]. Neurobiologically, global workspace theories emphasize frontoparietal cortical networks, particularly the prefrontal cortex, as the anatomical hub of the global workspace.

Empirical support comes from studies of masked priming, attentional blink, and binocular rivalry [20]. Disruptions to frontoparietal connectivity correlate with diminished consciousness, highlighting the importance of long-range communication between cortical regions. However, critics argue that the theory conflates consciousness with cognitive access and may not account for conscious experiences that lack global reportability, such as those occurring during certain meditative states or in cases of partial awareness [21].

### Integrated Information Theory

Integrated information theory offers a fundamentally different perspective by grounding consciousness in the intrinsic capacity of a system to integrate information [12]. Integrated information theory proposes that consciousness is defined by a system’s cause-effect structure and ability to generate more information than the sum of its parts. This integration is quantified as a mathematical measure of irreducible information.

The empirical neural correlates of integrated information theory focus on the “posterior cortical hot zone,” which includes parietal, temporal, and occipital regions. These areas are thought to possess the neuroanatomical properties required for high levels of integrated information. Unlike the other theories, integrated information theory emphasizes neural systems’physical structure and causal dynamics. Some tests that align with some of the commitments of integrated information theory have even shown promise [25].

Integrated information theory faces significant criticisms regarding its scientific validity, particularly through the”unfolding argument”[26]. This argument exposes a testability problem: integrated information theory claims that consciousness depends on specific causal structures that generate integrated information. However, any such structure can be mathematically”unfolded”into functionally equivalent networks with different causal architectures and information integration values. According to integrated information theory, these functionally identical systems would have different levels of consciousness—yet they would produce identical behaviors, reports, and experimental outcomes. This creates a falsification problem: if integrated information theory claims proposed conscious and non-conscious systems cannot be differentiated through any possible experiment, the theory becomes unfalsifiable and thus arguably unscientific. However, recent studies have made progress to show where integrated information theory has done better and worse than global workspace theories at predicting research outcomes^4^ [27].

#### Ethical Implications for Postmortem BIV Research

The target articles’ ethical protocols are consistent with traditional theories of consciousness. Higher-order theories and global workspace theories indicate that the lack of activity in the BIVs is good evidence that the BIVs are not conscious. We believe this is the case for integrated information theory, but since the theory focuses on mathematical and cause-effect properties of systems, it is less clear to say that no action potentials equal no consciousness. However, the empirical tests, PCIs, would certainly yield a negative result, indicating the BIVs are not conscious.

### Alternative Theories of Consciousness, Near-Death Experience

A few research lines raise doubts about our assurance of knowing whether any particular BIVs are conscious. This will slightly upgrade the justification of our precautions, but we do not imply that our precautions rely on them alone. Precaution is warranted in handling BIVs, even if traditional theories of consciousness are true. This is because we have historically failed on numerous occasions to know whether patients are conscious in certain conditions, severe brain injury [28], and cases of failed anesthetics [29], to name a few. However, plausible non-traditional theories of consciousness increase the precaution warranted. We will cover a few.

### Penrose and Hameroff Model

The first is the theory proposed by Penrose and Hameroff. The pair challenge the traditional view of the brain as a complex computer by suggesting neuronal network computations cannot fully explain consciousness and cognition. In more controversial terms, they claim consciousness is uncomputable [30]. Further, they propose that microtubules within neurons play a key role in consciousness ([31], 40, [32], 1871–72). The proposed mechanism is quantum resonances within microtubules. Specifically, the pair claims dipole oscillations in the superposition of benzene rings in microtubulin comprise a “qubit” and are responsible for consciousness ([33], 7–12). This, in turn, explains why anesthetics render individuals unconscious as they disperse these oscillations.

While this theory is still speculative and needs much more verification, the common reply that the human brain is too warm, noisy, and wet for consistent quantum effects ([34], chap. 10) is becoming less convincing. Despite proving nothing about the quantum effect’s connection to consciousness, a lab recently pointed to evidence of quantum effects in microtubules [35], at least establishing the possibility of the effects and at least questioning the most obvious defeater for the view. However, critics have raised substantial issues for the theory of quantum consciousness [36, 37].

The relevance of Penrose and Hameroff’s work should be obvious for our purposes. First, accepting the theory has some scientific merit means BIV research should seek to disperse these quantum effects. Barring any technique to monitor and disrupt such effects in the brain, constant administration of anesthetics to disperse these quantum effects seems warranted. Further, it questions our present methods for detecting consciousness, which do not assess for quantum effects. These tools, which depend on traditional theories of consciousness, may be inadequate for discerning conscious vs unconscious BIVs. However, there may be a significant, if not entirely, overlap between the methods of verifying traditional and alternative theories of consciousness. Of course, the lack of verification of a convergence raises doubt and the risk of harm. Research should investigate this possibility of convergence.

The even more worrying outcome is that Penrose and Hameroff are right; consciousness does depend on quantum effects, but in ways we don’t understand. If we must now explain what we consider a normal but complex computer with quantum mechanics, even more precautions may be warranted from our limited understanding of quantum mechanics. Such a Kuhnian shift warrants such a conclusion.^5^

### Near-Death Experiences

Research on near-death experiences provides more doubt as to our understanding of conscious experience in “abnormal” cases. Such experiences could be seen as brain dysfunction or adaptive hallucinogenic experiences (N 2024), evidence of quantum underpinnings of consciousness [38], or higher-dimensional brain-independent consciousness [39]. Some of these near-death observations are consistent with a Penrose-Hameroff model, but the evidence of why some near-death experiences occur under anesthesia [38] needs explaining.

A couple of considerations are entailed in reviewing the evidence of near-death experiences. The first is the risk of inducing near-death experiences in BIVs. Since subjects enrolling may be assumed to be terminally ill, they may experience one near-death experience, whether or not the subject enrolls in the study. Thus, such a risk is not added by the study and can’t be said to be caused by the study itself. The most troubling risk is that BIVs may experience prolonged or multiple near-death experiences from the brain resuscitation process and perfusion in BrainEx. One candidate cause of near-death experiences under the traditional model of consciousness is the naturally occurring DMT, the evidence being the convergence of self-reports of the phenomenon (N 2024). Researchers should take seriously the potential to induce near-death experiences in any BIVs. Consent models could outline opt-in/opt-out methods of preventing or allowing such experiences, should such control be available to researchers.

### Cellular Consciousness

Work by Michael Levin’s lab and collaborators has also given us cellular evidence [40–44] that may point to cellular consciousness. Of the three alternative theories to consciousness presented here, this is the most speculative. To be clear, this research is far from proving consciousness is cellular, and there are serious problems with such claims [45], and these researchers are not claiming they have done so. However, it at least raises the question of why such neuron-independent organismal agential functionings are not conscious. The reply “because it does not involve a neuron” is not entirely convincing. Perhaps the underpinnings of memory are more non-traditional than previously conceived because the underpinnings of consciousness are more non-traditional than previously conceived. This line of research admittedly needs more work to be precisely relevant for BIV research, as the most plausible theory of consciousness is one that depends on neurons.

Due to the profound possibility of BIV suffering, accommodating alternative but less plausible theories should be entertained so long as doing so does not risk previous precautions or is unduly costly. “Unduly costly” requires more unpacking and expertise than we can provide here, but we have examined alternative theories of consciousness to question whether our current precautionary standards are enough. We conclude we should consider these more alternative yet radical conceptions of consciousness and provide precautions for avoiding BIVW and mitigating harm in case we are already in BIVW. We will do so in Conclusion.

## Precautionary Principle

The PP has been inculcated by the European Union [46] and the United Nations [47]. The standard (or “weak”) Precautionary Principle holds that if an action or policy gives rise to plausible risks of serious or irreversible harm to human health or the environment, decision-makers should adopt anticipatory, cost-effective measures even in the absence of full scientific certainty [48]. It shifts the burden of proof onto those proposing the potentially risky activity to demonstrate it is safe rather than requiring opponents to prove it is harmful. Any precautionary measures taken must be proportional to the severity and plausibility of the potential harm and balanced against the prospective benefits and costs.

Less standard versions have been used to presume consciousness in related clades of organisms [49]. This same principle has been applied to brain organoid research: “If an organoid contains structures or mechanisms that any serious and credible theory of the human NCCs posits to be sufficient for conscious experience, we should take proportionate measures to regulate research on that organoid” [50]. Such an application of the PP is neither restrictive nor burdensome to research. It does not prevent research on organoids with potentially conscious brain regions, it merely requires precautionary measures to be applied to them. While the target article’s brains are not active, we have sufficient reason to believe that such a PP would require precaution for postmortem BIV research. More permissive brain research uses existing ethical standards in place for in vivo human brain development [51], the result being quite permissive for organoid research. However, such standards seem evidently to us to place much more caution in postmortem brain research.

Some work has already been done on the research of postmortem brain ethics. However, we believe much of the literature does not examine the potential for resuscitating brains and, therefore, takes the implicit stance that brain donation is tissue donation. While this is not a failure of the research at the time, the present article’s findings require revising or minimally reconsidering this question and whether or not the brain is conscious. Work has laid out the ethical concerns on postmortem brain research concerning consenting next of kin for research enrollment [52], postmortem privacy rights [53], brain biobanking [54], and consent [55]

There are many definitions of the PP [56, 57], 78). We take up the reasonableness and plausibility definition of the PP, which does not have to commit to ranking various values [56], 93). It is open to pluralism regarding values and uses a broad set of reasonable values, such as proportionality, fairness, consistency, and even other values from other moral theories. This pluralism could be a strength or a weakness. The best way to construe this definition of PP is that it is neither a rule from decision theory nor moral theory. The PP is open to pluralism regarding value and is still applicable to mitigate risk and maximize benefits. The PP also pays particular attention to potentially irreversible damage [57]. There are no necessary and sufficient criteria for this PP. It is more of a metarule that the statement can capture: take reasonable measures to prevent or mitigate plausible and serious threats.

This construal of the PP is helpful in decision-making under scientific uncertainty, where significant irreversible harm is a plausible outcome. Crucial to our discussion is recognizing that the PP provides an account of precaution, which is justifiable even for phenomena we don’t causally understand, such as consciousness. Even implausible theories should be taken seriously if the concomitant precaution is low cost. While it is an open question of what theories are beyond the pale of implausible and which are “reasonably” plausible, the PP allows us to consider a broad range of precautions when their costs are low.

We wish to invoke the epistemic elements of the PP while leaving the normative components aside. This means we will not discuss reasons for precaution regarding the uncertainty about normative truths. For instance, we will take for granted that the suffering of BIVs is bad. At present, we are only concerned with the precautions regarding uncertainty regarding whether or not negative mental states may occur in such experimental brains. Related to this interpretation of the PP is the commitment to shifting the burden of proof, many advocates of the PP suggest [48]. This burden of proof shift is even more compelling when high uncertainty and harm are involved. Given the inherent difficulty of assessing consciousness, the lack of behavioral evidence in cases of BIVs and organoids [50], and the significant risk of harm, researchers should accept the burden of proof for postmortem BIV research. This involves taking more implausible theories of consciousness seriously, especially if the predictions are low-cost.

However, the PP has detractors [58, 59]. The main objection often lodged at PPs is that a strong interpretation leads to contradicting recommendations and paralyzes our decision-making. Sunstein argues that a strong PP is committed to “regulation […] whenever there is a possible risk to health, safety, or the environment, even if the supporting evidence remains speculative and the economic costs of regulation are high” [59], 24–31). The obvious response is to deny the efficacy of the strong version of the PP, which we recommend. Further, authors have argued that such an interpretation of the strong PP presumes all risks invoke a PP and that risks of action and inaction are equal [60], 20). If we deny this, it appears that this objection has been avoided.

Another significant drawback to the PP we are applying is that it is hard to distinguish it from the practical reasoning inherent in any decision-making process [61]. For instance, one may think that cost–benefit analysis is presupposed in any PP, therefore, the PP is redundant.^6^ This is further complicated by the fact that reasonable precaution depends on what moral principles we endorse. Our version of the PP is context-sensitive, and perhaps a consistent set of precaution proscriptions is challenging to find. Since the PP admits to pluralism regarding moral principles, under one set of principles, one precaution may seem reasonable, while under another, it may seem unreasonable. Especially under a weaker interpretation, which we are committed to. However, more substantive moral principles may bolster the PP. This is because the PP itself may not provide sufficient guidance for determining the proportionality of harms to benefits or the fairness of their distribution. Thus, other frameworks could assist with this assessment. For example, utilitarianism [62], 120–31) might help conceptualize proportionality, and Rawlsianism might help conceptualize justice [63] within a PP framework.

Further objections [48] and replies have been made [64–66], but we can not rehash everyone here. The primary motivation for using the PP in BIV research is the severity of the risks BIVW poses in light of alternative theories of consciousness. Without the PP, such theories may not be given enough credence in typical IRB or animal committee approval of studies. Decision theory may struggle to develop any reasonable probability to assign such alternative theories of consciousness and thus fail to account for it sufficiently.

Here we employ a weak form of the PP. This version recognises that plausible, severe, and potentially irreversible harms, such as unintentionally subjecting BIVs to suffering, justify shifting the burden of proof to investigators. Crucially, however, the requirement is for proportionate, cost-effective precaution, not for halting research outright. The aim is to protect against the worst-case scenario without unduly constraining the best-case.

## Why the PP and not Expected Utility Theory or Maximin

We can not be sure BIVs are not conscious. Deprived of sensations, even hallucinations or instances of phantom pain may not occur. This is entirely an open question. Thus, what precautions are necessary are unknown. However, recent advancements in empirical evidence for consciousness make determining the existence of conscious states without behavioral correlates [28] more feasible. Directly translating such research to brain tissue outside of a body faces some problems [4], as already mentioned. After all, such patients still have a brain attached to sensory components. We also don’t know how “minimal” consciousness will be for BIVs, and this uncertainty justifies high-level precautions. Further research into the underpinnings of consciousness is warranted.

Given this uncertainty and risk, the PP is the most appropriate application to BIV research. Expected utility theories differ from the PP in several relevant ways. First, unlike expected utility theory, the PP does not require calculable probabilities and utility for decision-making [56].^7^ This is helpful in medical research settings, as quantifying the utility or probability of mental states is troublesome. Thus, the expected utilities’ obvious usefulness in economic domains. Expected utility theory faces a huge epistemic problem for application in BIV research. Calculating how probable alternative theories of consciousness face huge uncertainty. Thus, calculating expected utility is difficult for BIV research.

The PP is a better approach than decision theory because the most appealing strand for BIV research, maximin, seems overly cautious when applied. Our application of the PP can avoid such overapplication of precaution. Maximin gives too much credence to implausible risks with adequate precaution. Maximin seeks to raise the floor (worst outcomes) rather than the ceiling (best outcomes). The PP, conversely, takes precautions against implausible risks but does not prohibit taking the risk to gain a benefit. Further, it can consider increasing the best possible outcomes as a reason to accept some disaster risk, unlike maximin. Maximin may forestall any BIV research since the ability to know that BIVs have no experience is too high a bar to establish. Even if we deny the need to give a convincing reply to the problem of other minds, maximin seems to be committed to preventing BIVs from being unable to prevent their own suffering. This, in turn, seems to be committed to having a way to communicate with BIVs and being able to terminate the experiment at their request. This will require giant neurotechnological advancements, and awaiting such technology will forestall BIV research indefinitely. We are open to criticisms of this critique of maximin, but we also ask maximin advocates to address how to calculate the plausibility of alternative theories of consciousness, perhaps a larger worry for maximin for BIV research.

Our PP applied to postmortem BIVs also avoids seeming over-applied, as is the case in brain organoids. Authors have invoked the PP against others to conclude that treating all brain organoids as if they were conscious is the best path forward [5]. We agree that some of the outcomes of the PP seem overapplied when considering single-digit clumps of neural organoids [4]. However, we find the general principle quite warranted for postmortem BIVs. Treating all postmortem BIVs as if they were conscious should be more attractive to those criticizing the PP for over-precaution in brain organoids. For the reasons already expressed, the evidence is much more robust in accepting this outcome of the PP for postmortem BIVs and not brain organoids.

Many ethical-legal questions await further interrogation, which we can not get into here. One worth mentioning that will seriously increase or decrease the precautions justified by the PP are the mortality and, thus, the patient status of the BIV. If postmortem BIVs are considered patients, there will be very little tolerance of IRBs for BIV research, mainly because there will be no clinical benefit to the patient. Conversely, if BIVs are considered donated organs, the real threat of ending up in BIVW increases, as researchers have more discretion with donated organs than patients.

## Practical Precautions

Some precautions fall out of applying the PP to prevent bad mental states and maximize positive mental states in BIVs. Should postmortem BIV research advance to human trials, we have some obvious general design recommendations to be followed by more specific recommendations.

The obvious first general precaution is to limit sample size and continually research potential risks. This approach is similar to novel Phase I studies with uncertainty and risk, a method of simple harm reduction [67]. In this slow research phase, ethical problems like monetary incentives distorting ethical judgments [68] can be addressed more quickly and easily. The hope is to allow a slow progression to avoid ethical disasters such as the TeGenero trial [69].

Of course, the PP advocates for more research to understand the possibility of minimal consciousness, alternative consciousness, etc., relevant to potential BIV experiences. Minimal consciousness [70] concerns the “what kind” question mentioned earlier. It may test our best empirical tools for assessing it, with the additional problem of translating such evidence in embodied [28] brains to BIVs [4]. This commitment removes the burden of proof shift [48], mentioned earlier, requiring researchers to provide evidence supporting their protocols.

We now move to more specific precautions to raise the ceiling and floor of potential BIV experience.

### Anesthetics

Anesthetics are a prime candidate for preventing negative mental states in BIVs. The target article even had anesthetics ready to use if brain activity indicating consciousness occurred. However, we recommend constant anesthetic use during the experiment. This is to hedge against quantum theories of consciousness being true and our current measures of consciousness not being perfectly correlated with the activity of the quantum effects.

There are a few potential side effects of using anesthetics on BIVs. Assuming BIVs are capable of being conscious, long-term anesthetic use may lead to anesthetic tolerance and recovery of consciousness. If consciousness tests fail to detect consciousness regaining in such a BIV model, BIVW will become true. Further, anesthetics may be ineffective at doses used or ineffective for reasons we don’t understand. Lacking behavioral evidence or self-reporting in a typical clinical setting could lead to unintentionally causing nausea or distress to the BIV, causing more significant harm than if nothing had been administered to the BIV.

### Cingulotomies

There are no pain receptors in the brain. However, with the affective systems for pain residing in the brain, it is worth considering whether BIV activity correlates with typical pain. Plenty of preventive measures ensure such activity is unlikely. However, for the reasons mentioned above, further caution is warranted.

Assuming the research goals are not associated with pain,^8^ cingulotomies could be adopted to prevent pain [71]. Opioids have been documented to achieve a similar behavioral output from the disconnect between sensory and affective systems related to pain [72–74]. Cingulotomies achieve this outcome via surgical means. Cingulotomies are still used to manage intractable and pharmacologically unresponsive pain or depression today. The procedure is simple and involves lesioning the affective pain areas of the brain. MRI would confirm the successful lesioning of the appropriate areas. While MRI use increases the cost of such research, such assurance against extreme pain seems well justified.

Cingulotomies may be a long-term solution but are not permanent [75]. It is unclear if a BIV could recover from a cingulotomy, but this is a question researchers should consider if their experiment lengths exceed the efficacy length of cingulotomies. Side effects of cingulotomies have been reported, such as nausea and headaches, but these side effects are typically short-term and infrequent [76].

### Opioids

While opioids may be short-acting, serious considerations need to be made in light of the possibility of conscious BIVs having negative experiences that the administration of opioids may prevent. If this is the case, the PP may advise considering the administration of opioids not only to foster a positive experience but also to prevent negative experiences. Of course, opioids work by modulating neuronal activity. However, it is a worthy precaution, given that we don’t know if the experimental measurement of neuronal activity completely overlaps with actual neuronal activity.

Presuming a euphoric state is necessarily a positive for all patients is unwarranted. However, it is unclear whether any BIV experiences could be positive. Disembodied experiences will be foreign to the BIV. We have no scientific evidence that such experiences wouldn’t eventually be distressing. Therefore, the PP advises us to expect any BIV experience to be negative without serious pharmacological intervention.

The specific mental states we wish to prevent are social isolation and boredom. Opioids play a clear role in the sensation of social affiliation and bonding [77].^9^ Social isolation is considered torture and causes brain damage [78]. Plausibly, constant administration of opioids to BIVs may lessen such negative experiences. Since there is no real worry of addiction^10^ or the possibility of death from breathing depression, opioids may be an appealing precaution for BIVs.

There are substantial issues, however, with prolonged administration of opioids in BIVs. First, as is the case with anesthetics, self-report and behavioral evidence readily available in palliative and clinical care is not available for researchers experimenting on BIVs. Researchers should examine the best administration methods to prevent the brains from withdrawing, which would require knowledge of drug resistance, sensitivity, and tolerance. Numerous adverse side effects of opioids, such as nausea or distress, could confound any positive impact on the long-term well-being of BIVs. Long-term opioid use could also complicate a research model in numerous ways, ranging from effects on mood, neurogenesis, pain perception, and memory [79–82], to name only a few. For these reasons, opioids are likely only advisable for short-term BIV experiments. This approach could avoid the concerns of withdrawal and tolerance.

Admittedly, our precautions do not prohibit BIV experience. However, we believe such precautions raise the floor and ceiling in a manner consistent with the PP. If BIVs are somehow conscious, the precautions are clearly warranted. For instance, perhaps a BIV experiences a hallucination despite all of the precautions. It is realistic to expect that such an experience may be characterized as negative, but cingulotomies and opioids may entirely negate the negative affective element away from much of the experience [72]. BIVs may still have negative experiences. Such strange, confusing, or shocking experiences may occur, but such experiences are likely better without affective sensations of pain or potential pain and a feeling of drug-induced euphoria. Simply relying on anesthetics leans too heavily on one preventative method for preventing negative experiences. Similarly, phantom limb pain may be managed with complementary interventions. If anesthetics fail to prevent phantom limb pain, both cingulotomies paired with other procedures [83] and opioids [84] independently have shown such overlapping, yet imperfect, efficacy and therefore offer superior precaution than mere anesthetics.

We believe the PP recommends a minimum of cingulotomies for postmortem BIV research. Such a precaution is not overly restrictive or burdensome for research, but provides quite a minimal floor for BIV experience. While arguments have been raised that argue the PP is excessively restrictive and unscientific [65], the precautions we raise here do not forestall present BIV research by advocating for the precautions at hand.

#### IRBs, Person Status of Postmortem Brains, and Consent

IRBs can play a critical role in postmortem brain research. In any area of research where a high level of uncertainty of harm exists, IRBs face difficult decisions that may either restrict researchers or risk harm. IRBs should consider the above precautions and the time frame of experiments. Some precautions make more sense at different time scales and cause less concern for adverse effects when experiments are shorter. For instance, worries about boredom or social isolation can be ignored and considered unreasonable in short-term experiments. It will be up to the IRBs’judgment to determine if the proposed precautions are reasonable over the experiment’s period. IRBs should also improve oversight by appointing members with expertise in neuroscience or relying on outside experts with such expertise. IRB members will likely not have the expertise in pharmacology required to assess the suitability of anesthetics or opioids should they be used as precautions. By staying in contact with neuroscience researchers at their institutes, they can keep informed about research that may pose issues.

There is a looming metaphysical problem with postmortem brain research. We cannot do it justice here, but we will mention it, as it may be open to IRB oversight. More problems are associated with informed consent and brain tissue research [55]. However, the main problem we will gloss is whether the brain is a subject or a donor.

A problem with the interpretation that the brain is a person who is a subject in an experiment is that the person consenting to the experiment may not be the same person as the brain during the experiment. There are obvious implications to answering in either way. One person enrolling in an experiment cannot bind another person to continue to be enrolled. Therefore, if it is the case that BIVs are different persons, they can not ethically be researched unless they consent. If the BIV is considered the same person who consented to the study, informed consent may allow the person to consent to the potential harms they may face as a BIV. This could include not being able to stop the experiment despite wishing to do so. However, discontinuation protocols may be in put in place if the brains appear conscious.

In comparison, there are far fewer issues to resolve if researchers take the BIV to be brain tissue that has been donated. So long as it is reasonable to believe that the BIV is not conscious, IRBs may be satisfied with this conclusion. However, cingulotomies may reassure all involved in such research.

## Conclusion

Many more mental states could be categorized as “unwanted” or “bad” by BIVs. We can not cover all of them, but more research should be done to evaluate them. The assumption that sensory input is required for conscious experience needs further research before the widespread application of postmortem BIVs. The paper has provided a clear indication that postmortem BIVs present a clear leg up on organoids as a plausible model of disembodied consciousness that critics of organoid consciousness have raised [4]. As such, we have provided general and specific precautions that future BIV researchers should seriously consider to raise the ceiling and floor of potential BIV experience. Our precautions emanating from the PP hedge against the reasonable expectation that non-traditional theories of consciousness are true. These precautions, however, are more permissive than the overapplications of the PP we have mentioned in brain organoids.

## Footnotes

1 A similar ethical dilemma faces Cheng Xin’s staircase project in the fiction trilogy *Remembrance of Earth’s Past* where Yun Tiaming’s brain is shot into space to gather information about the approaching alien force [85]. The consent for such an endeavor included the potential for capture and extended, perhaps unending, pain, or simply no experience.

2 Maximin is a rule for making decisions under ignorance; that is, when we cannot assign probabilities to the outcomes associated with different options [56]. Maxim instructs us to choose the option with the best worst possible outcome.

3 Some authors have pointed out that the temperature used in the experiment is too low to qualify as normothermic [86].

4 Particularly, integrated information theory has seemed to been right about shifts in conscious states requiring brain activity at the back of the brain, rather than the front, which global workspace theory has emphasized, seemingly wrongly.

5 Of course, it could turn out consciousness is explained by already understood quantum effects. We leave such a comment to the physicists.

6 Thanks to Stephen Kershnar for this objection.

7 Expected utilities theory applies to situations in which we can assign probabilities and utilities to outcome associated with different options. The theory instructs us to choose the option with the maximum expected utility, which is a product of probability and utility. For example, if I am deciding whether to spend $1 to buy a lottery ticket for a chance at winning $100, and my odds of winning are 1/500, then my expected utility would be 1/500 × $100 = $0.20. Thus, according to this theory I should not buy the ticket because I would have a 100% probability of losing $1, for a net of $0.20—$1 = −$0.80.

8 If a study is not intending there to be consciousness in a model but wants to study pain, it is more appropriate to say they are studying nociception.

9 This explains popular music sayings. “There’s a feeling you’re replacing embrace,” (1975 2018) and “You can’t help me now, you guys/And all you sweet girls with all your sweet talk/You can all go take a walk,” [87] and “Heroin, it’s my wife and it’s my life,” [87].

10 Such a conversation may be had with palliative care doctors, where an opioid-dependent dying patient is reminded withdrawal is no concern for those who never stop taking opioids.
